# Supplementary material for: High-resolution experimental and computational electrophysiology reveals weak β-lactam binding events in the porin PorB
Source: Sci Rep. 2019 Feb 4;9:1264. doi: 10.1038/s41598-018-37066-9 (PMC6362148; doi:10.1038/s41598-018-37066-9)
Supplement: Supplementary file 1 — Supplementary information [file 41598_2018_37066_MOESM1_ESM.pdf]

**SUPPORTING INFORMATION**

# High-resolution experimental and computational electrophysiology reveals weak β-lactam binding events in the porin PorB

Annika Bartsch,[1,&] Salomé Llabrés,[2,&] Florian Pein,[3] Christof Kattner,[4,§] Markus Schön,[1] Manuel Diehn,[3] Mikio Tanabe,[5] Axel Munk,[3,6] Ulrich Zachariae,[2,7,*] Claudia Steinem[1,*]

[1] Institute of Organic and Biomolecular Chemistry, University of Göttingen, Tammannstraße 2, 37077 Göttingen, Germany,

[2] Computational Biology, School of Life Sciences, University of Dundee, Nethergate, Dundee DD1 5EH, UK

[3] Institute for Mathematical Stochastics, University of Göttingen, Goldschmidtstraße 7, 37077 Göttingen, Germany,

[4] ZIK HALOmem, Membrane Protein Biochemistry, Martin-Luther-University Halle-Wittenberg, Kurt-Mothes Straße 3, 06120, Halle (Saale), Germany,

[5] Institute of Materials Structure Science, Structural Biology Research Center, KEK/High Energy Accelerator Research Organization, 1-1 Oho, Tsukuba, Ibaraki 305-0801, Japan

[6] Max Planck Institute for Biophysical Chemistry, Am Fassberg 11, 37077 Göttingen, Germany

[7] Physics, School of Science and Engineering, University of Dundee, Nethergate, Dundee DD1 4NH, UK

[§] Current address: Juno Therapeutics GmbH, Göttingen, Germany

[&] Equal contribution

* Correspondence to: u.zachariae@dundee.ac.uk or claudia.steinem@chemie.uni-goettingen.de

**Electrophysiological setups and measurements.** Single channel recordings of PorB were performed on solvent-free planar bilayers using the Port-a-Patch (Nanion Technologies, Munich, Germany). Giant unilamellar vesicles (GUVs) composed of 1,2-diphytanoyl-*sn*-glycero-3-phosphocholine (DPhPC)/cholesterol (9:1) were prepared by electroformation (AC, $U$ = 3 V, peak-to-peak, $f$ = 5 Hz, $t$ = 2 h) in the presence of 1 M sucrose at 20 °C.[1] Spreading of a GUV in 1 M KCl, 10 mM HEPES, pH 7.5 on an aperture ($d$ = 1-5 µm) in a borosilicate chip by applying 10-40 mbar negative pressure resulted in a solvent-free membrane with a resistance in the GΩ-range. Once the membrane with a GΩ–seal was formed, varying amounts of a PorB stock solution (2.2 µM in 200 mM NaCl, 20 mM Tris, 0.1 % (*w*/*w*) N,N-dimethyldodecylamine N-oxide (LDAO), pH 7.5) were added to the buffer solution (50 µL) at an applied DC potential of +40 mV. Current traces were recorded at a sampling rate of 10 kHz and filtered with a low-pass four-pole Bessel filter of 1 kHz using an Axopatch 200B amplifier (Axon Instruments, Union City, CA, USA). For digitalization, an A/D converter (Digidata 1322; Axon Instruments) was used and data analysis was performed with the Clampfit 10.4.0.36 from the pClamp 10 software package (Molecular Devices, Sunnyvale, CA, USA). Solvent-free bilayers were used to obtain the three conductance values of the PorB trimer (**Fig. S1**), as the PorB channel gates much more frequently than in black lipid membranes (BLMs) (**Fig. S2**).

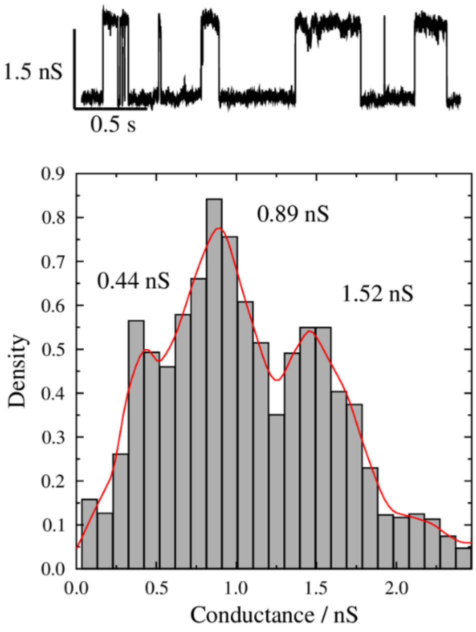


**FIGURE S1.** (**Top**) Representative single channel conductance traces of PorB in solvent-free membranes (DPhPC/cholesterol, 9:1) recorded at +40 mV in 1 M KCl, 10 mM HEPES, pH 7.5. (**Bottom**) Event histogram of the conductance levels including kernel density estimations using a Gaussian kernel (red line). The conductance states found for PorB ($n$ = 5269) are $G_M$ = (0.44 ± 0.18) nS, $G_D$ = (0.89 ± 0.26) nS and $G_T$ = (1.52 ± 0.46) nS. Indicated errors are the FWHM.

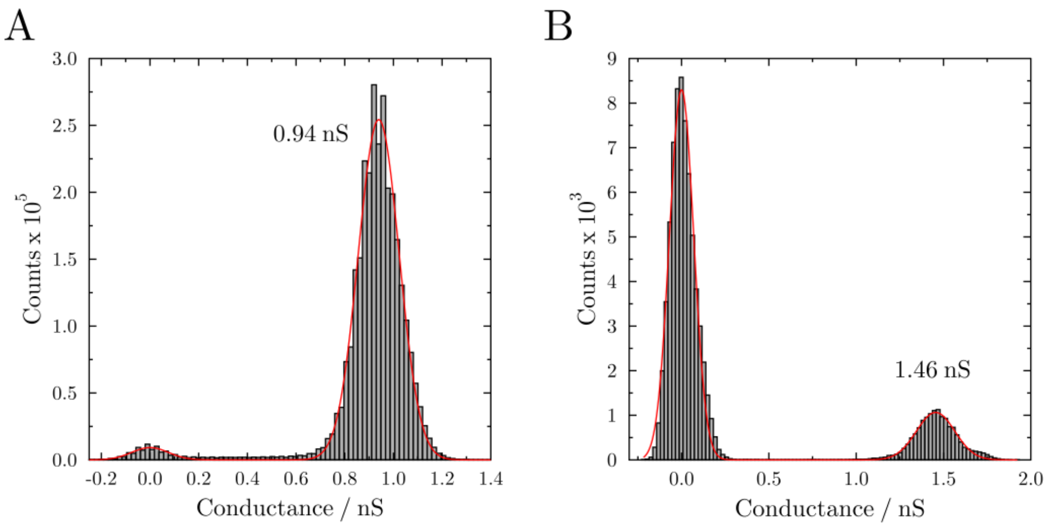


**FIGURE S2.** Open probability of PorB in black lipid membranes and solvent-free membranes. Point amplitude histograms of PorB measured in (**A**) a black lipid membrane and (**B**) in a solvent free bilayer at +40 mV. In both cases, only the whole trimer showed gating. The red solid lines are Gaussian distributions fit to the histogram peaks. The open probability was calculated from the Gaussian fitted areas of the two observed peaks. In black lipid membranes an open probability of 97% was found, whereas in solvent-free bilayers the open probability was only 21%.

For electrophysiological measurements in the presence of ampicillin including the corresponding control experiments, BLMs were used. BLMs were prepared by adding 1-2 µL of lipids (DPhPC/cholesterol, 9:1) dissolved in *n*-decane (30 mg/mL) to an aperture (*d* = 50 µm) in a PTFE foil (DF100 cast film, Saint-Gobain Performance Plastics, Rochdale, Great Britain) fixed between two cylindrical PTFE-chambers filled with 3.0 mL buffer (1 M KCl, 10 mM HEPES, pH 7.5 or pH 6). Protein was added to the *cis* chamber and inserted by stirring at an applied DC potential of +40 mV. After protein insertion, ampicillin was added from a stock solution (25 mM in 1 M KCl, 10 mM HEPES, pH 7.5 or pH 6.0) to both sides of the BLM. For control experiments, ampicillin was added only to the *trans* side. Current traces were recorded at a sampling rate of 50 kHz and filtered at 5 kHz.

**Analysis of current traces in the presence of ampicillin.** Model-free idealizations are obtained by JULES.[2] Its combination of multiresolution techniques and local deconvolution allows a precise idealization of events below the filter length, in particular amplitudes and residence times that are smoothed by the filter are reconstructed with high precision (**Fig. S3**). In this manner, JULES extends the model-free idealization method JSMURF[3] to scales below the filter length. The idealized blockage supports a two state Markov model. The conductance losses by blocking a single channel are determined by Gaussian fits of the amplitudes. Residence times and frequencies are determined by fitting the Markov model taking missed events into account. An analysis with a new Hidden Markov model approach, which is able to take the lowpass Bessel filter explicitly into account, confirms these results.

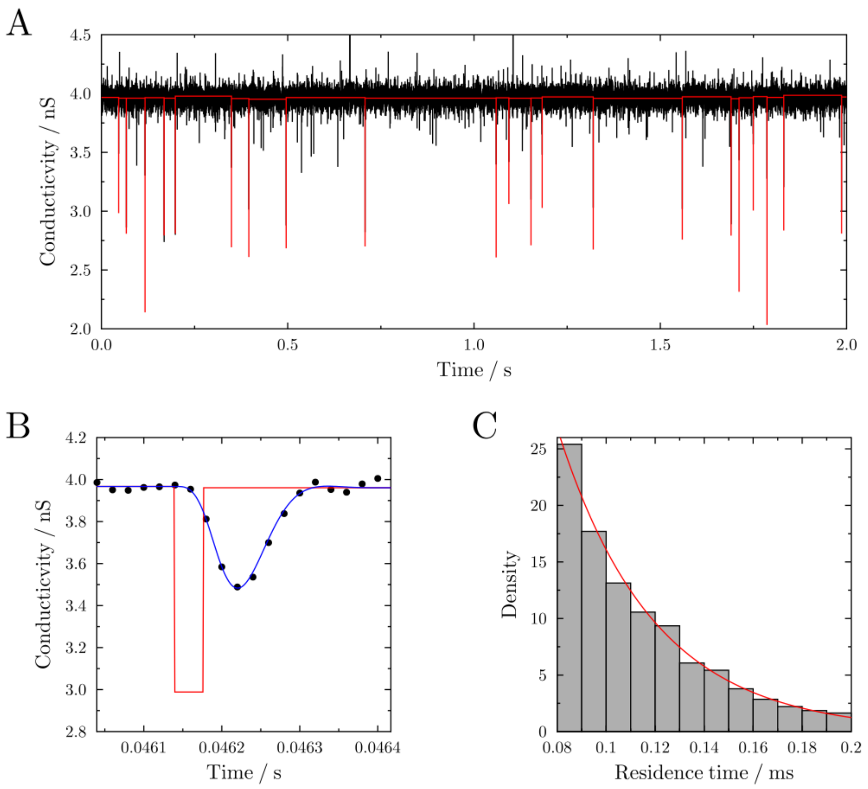


**FIGURE S3.** (**A**) Signal reconstruction of a conductivity-time trace using JULES; (**B**) shows a zoom-in of the trace shown in (**A**). Data points are presented in black and the reconstructed signal in red. After filtering, this reconstruction (blue line) fits the data well. (**C**) Histogram of the residence time extracted from the conductivity trace shown in (**A**). The exponential fit (red line) takes missed events into account and is rescaled such that the areas under both plots are one.

**Technical details of the statistical analysis**

**Idealizations.** Model-free idealizations are obtained by JULES (JUmp Local dEcovolution Segmentation filter)[2] by calling the R-function jules in the package clampSeg (https://CRAN.R-project.org/package=clampSeg) with default parameters. Its combination of multiresolution techniques and local deconvolution allows a precise idealization of events below the filter length, in particular amplitudes and residence times that are smoothed by the filter are reconstructed with high precision (**Fig. S3B**).

For comparison, idealizations using a discrete time Hidden Markov model (HMM) taking into account the filter are obtained. The Markov assumption is confirmed (see below). In a pre-processing step, artifacts caused by the electronics, fluctuations on large time scales and outliers are removed. For the HMM the following parameters are used: A digital filter of length six approximating the used Bessel filter, the amplitude found by JULES,[2] a fluctuating base line given by local medians and a global variance computed by sdrobnorm (stepR package, https://CRAN.R-project.org/package=stepR). A new forward algorithm[4] is used to learn the transition matrix based on a likelihood approach. The final idealizations are obtained by a Viterbi algorithm. In order to cope with the discrete filter, both procedures use a meta state space, resulting in $2^6$ states. Code is available on request.

**Missed events.** Simulations show that JULES detects events with residence times of at least 80 µs with probability almost one at the worst signal to noise ratio. Hence, the analysis is based on detected events between 80 - 200 µs, since some shorter events are missed and some larger events might be rare gating events of the channel itself and not ampicillin blockage.

The event histograms of the idealized amplitudes, i.e., the difference of the conductance levels of the open and blocked channel, show one well-separated peak not found in the absence of ampicillin (**Fig. 1**). All other events are neglected as they cannot be associated with the ampicillin blockage. For visualization, additional kernel density fits with Gauss kernel and bandwidth 0.05 by the R-function bkde in the package KernSmooth (https://CRAN.R-project.org/package=KernSmooth) are shown. The conductance losses by ampicillin blockage are determined by Gauss fits.

The HMM approach is able to detect events with residence time of at least 20 µs corresponding to one sample point, but detects in addition artifacts of a length of one and two sample points. Therefore, only events of at least three sampling points in length are included in the analysis. To compensate for this, for both approaches the later analysis has to take into account missed events.

**Confirmation of the Markov model.** The events detected by JULES with a length between 80 - 200 µs are used to explore the dwell times, i.e., the residence times and the times between two blockage events. Their occurrence decreases exponentially in their duration, see exemplary the histogram of the residence

times in **Fig. S3C**. Note that for visualization purposes the exponential fit is rescaled such that the surface under the curve in the shown range is one as it is for the histogram. Moreover, **Fig. S4** shows uncorrelated dwell times. Together with the exponential decay this supports a Markov assumption.

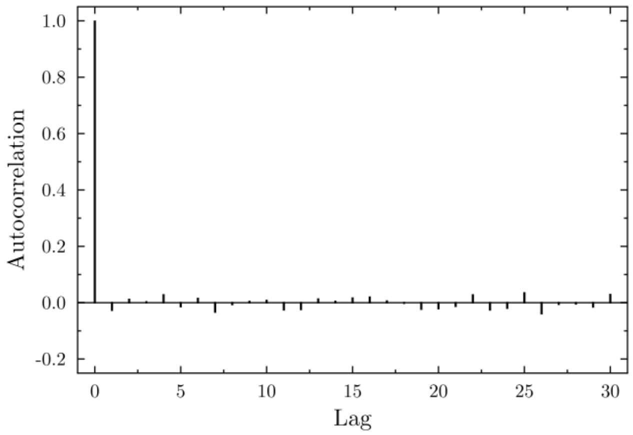


**FIGURE S4.** Empirical autocorrelation between the idealized dwell times, i.e., residence times and time between two events. The correlations close to zero support a Markov model.

**Residence times and frequencies.** The average residence time is calculated for both approaches via maximization of their corresponding likelihood assuming an exponential decay and a missing of events as described above. Maximization is performed by the Broyden–Fletcher–Goldfarb–Shanno (BFGS) algorithm,[5] using the R-function optim. The Markov model assumption allows determination of the ampicillin blockage frequency for a single channel. To this end, the observed distances between two events are multiplied with the probability that an event is not missed and with the number of channels. The frequencies are obtained by the inverse of the mean of these rescaled times. The number of channels is calculated by dividing the conductance without blockage by the amplitude of a blockage, both determined by Gaussian fits.

Additionally, 95%-confidence intervals are computed for the residence times (**Fig. S5A**) by a normal approximation and for the frequencies (**Fig. S5B**) by an exact confidence interval. For illustration, we report standard deviations for the averaged residence times and frequencies (**Fig. S5A**), but note that no significance statements can be drawn from these intervals. We stress that the averaged residence times and frequencies based on JULES and the HMM approach were always close to each other and lead in all cases to the same conclusions, as illustrated in **Fig. S5**.

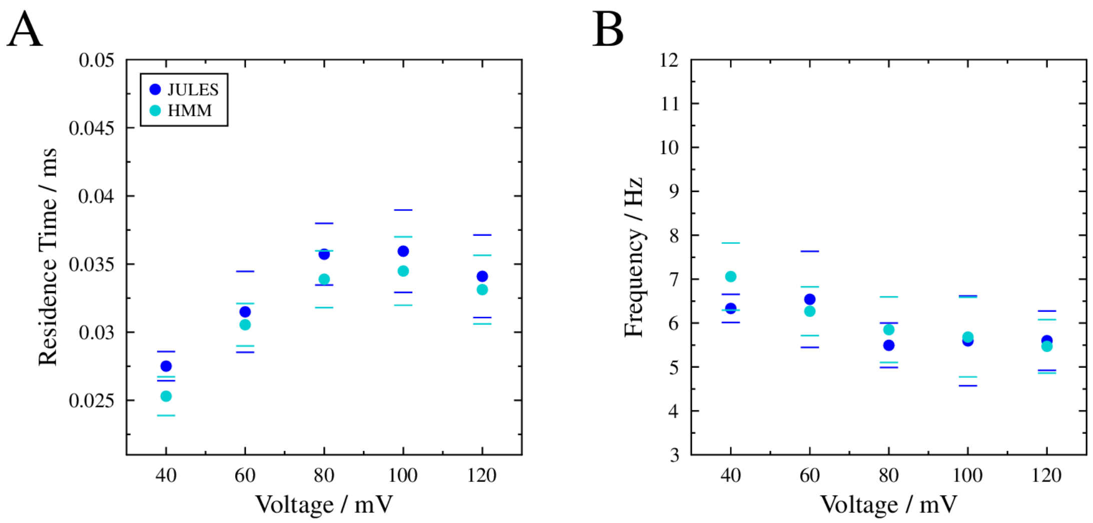


**FIGURE S5.** (**A**) Voltage dependent residence times and (**B**) blockage frequencies of PorB at pH 7.5, determined using JULES and a hidden Markov model (HMM) in comparison. $n$ = 4 measurements were evaluated and averaged. The error bars represent the standard deviation.

**Docking calculations.** The binding mode of ampicillin was explored by means of docking calculations carried out with GOLD[6] and rDock[7,8] software packages. The structural model of PorB considered in the docking calculations was the X-ray structures of the PorB solved by Kattner et al. (PDB entry 3VY8).[9] Both zwitterionic and anionic forms of ampicillin were subjected to 100 docking runs. Whereas the protein was kept rigid, GOLD and rDock account for the conformational flexibility of the ligand during docking calculations. Resulting binding poses were analyzed by visual inspection in conjunction with the docking scores. The docking poses were ranked using Molecular Mechanics Poisson-Boltzmann Surface Area (MMPBSA) approach using a salt concentration of 1M and the internal dielectric and external dielectric constants set to 80.0 as described in Homeyer N et al[10,11].

**System set up.** PorB was modelled using the X-ray structure obtained by Kattner et al. (PDB entry 3VY8).[9] PorB trimers were embedded into a preequilibrated 160×160 Å$^2$ 1-palmitoyl-2-oleoyl-*sn*-glycero-3-phosphocholine (POPC) bilayer accounting for 942 POPC molecules. A 25 Å layer of SPC/E water molecules was set up at both sides of the bilayer, and $Na^+$ and $Cl^-$ were added to achieve an ionic strength of 1 M. GROMACS utility membed[12,13] was used to embed PorB trimers into a POPC bilayer. The aqueous solution consisted of about 57000 water molecules and 1866 $Na^+$ and 1896 $Cl^-$ ions in both the apo and ampicillin bound system. The Parm99SB force field,[14,15] and virtual sites for hydrogen atoms[16] were used for the protein. The POPC molecules were parameterized according to the lipid parameters derived by Berger et al.,[15,17] the SPC/E water model was used to model water molecules[18] and Joung and Cheatham parameters[19] were used to model the counter ions. The zwitterionic ampicillin molecule was parameterized using the gaff force field[20] in conjunction with RESP (HF/6-31G(d)) charges[21] as implemented in the Antechamber module of the AMBER12 software package.[22]

**Molecular dynamics simulations.** Molecular simulations were carried out with the GROMACS molecular dynamics package, version 5.1.5.[23] For each system, the geometry was minimized in four cycles that combined 3500 steps of steepest descent algorithm followed by 4500 of conjugate gradient. Thermalization of the system was performed in 6 steps of 5 ns; each step gradually increased the temperature form 50 K to 320 K, while the protein was restrained with a force constant of 10 kJ mol$^{-1}$ Å$^{-2}$. The systems were equilibrated for 100 ns keeping the protein restrained. Production runs accounted of 200 ns-long trajectories. The temperature was kept constant by weakly coupling ($t$ = 0.1 ps) the membrane, protein, and solvent separately to a temperature bath of 320 K with the velocity-rescale thermostat of Bussi et al.[24] The slightly higher temperature was chosen to ensure that the membrane remains in a fluid phase. The pressure was kept constant at 1 bar using semi-isotropic Berendsen coupling.[25] Long-range electrostatic interactions were calculated using the smooth particle mesh Ewald method[26] beyond a short-range Coulomb cut-off of 10 Å. A 10 Å cut-off was also

set for Lennard-Jones interactions. The LINCS algorithm[27] was used to restrain the system and the SETTLE algorithm[28] was used to constrain bond lengths and angles of water molecules. Periodic boundary conditions were applied. Taking advantage of the Berger lipid model and the virtual sites, the integration time-step was set to 4 fs.

**Computational Electrophysiology simulations (CompEL).** Each system was duplicated along the *z*-axis to construct a double bilayer system, and ionic imbalances from 4, 8 and 12 $Na^+$ were used between the aqueous compartments to generate a range of transmembrane potentials as from ± 130, ± 350 and ± 500 mV, as previously described by Kutzner et al.[29] Production runs accounted of 200 ns-long trajectories for the apo systems and 400ns-long trajectories for the ampicillin-bound systems. The applied membrane potential was calculated using the GROMACS utility gmx potential overlapping 20-ns time windows. Following the protocol outlined in Kutzner et al.[29], conductance (G) calculations (**G = I/ΔU**) were based on the observed ion current **I**, as a function of the potential difference ΔU between the compartments. Since potential and flux are time-dependent, ΔU(t) and **I**(t) were determined within 20-ns time windows (with a 10-ns overlap among consecutive windows) in the case of apo PorB and within 50-ns windows (with 25-ns overlap among consecutive windows) for ampicillin bound PorB simulations. The longer windows in the latter case were used in order to improve sampling in these lower-conductance situations.

**Analysis of the molecular dynamics trajectories.** MDAnalysis[30] and MDtraj[31] were used to analyze RMSD, distances, flux of water molecules and computational conductance values.

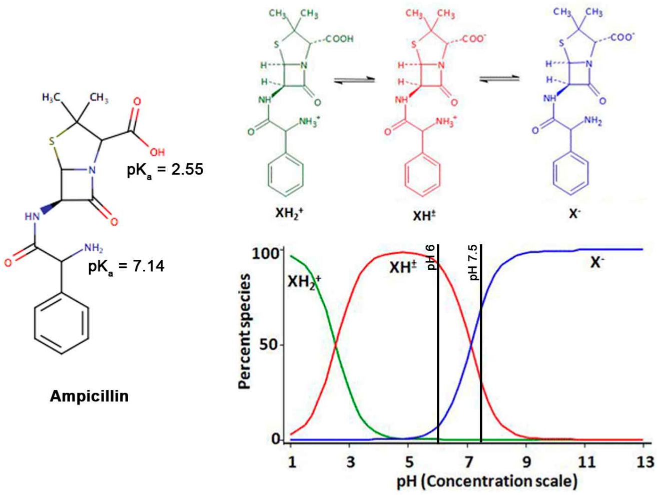


**FIGURE S6.** Protonation states of ampicillin at the experimental pH values. At pH 6 the $XH^{\pm}$ form is 94.3 and the $X^-$ 5.7 % of the total ampicillin population. At pH 7.5 the $XH^{\pm}$ form is 40.3 and the $X^-$ 59.7 % of the total ampicillin population.

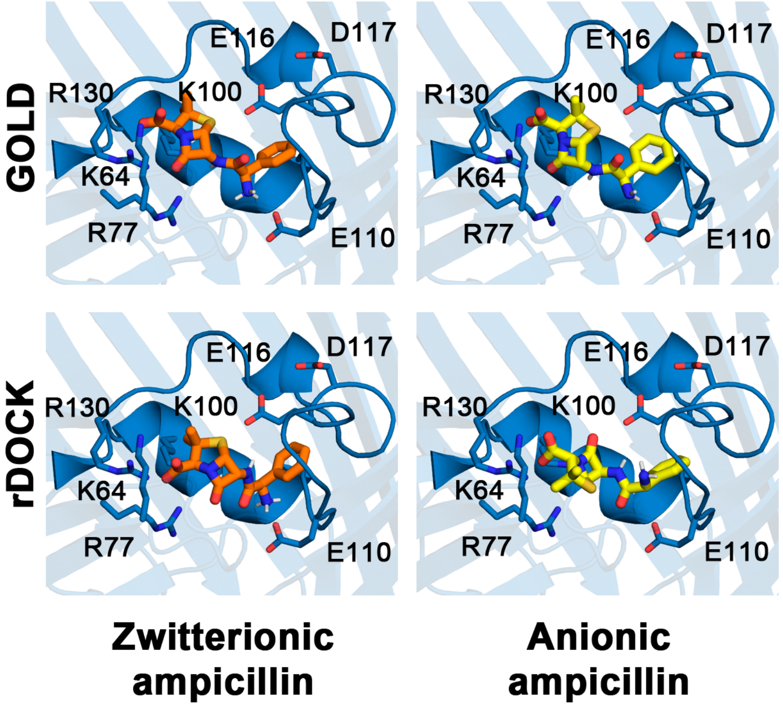


| GOLD | Pose | Score | PLPScore | hbond |
|---|---|---|---|---|
| **Zwitterionic ampicillin** | 1 | -57.24 | -38.84 | 6.53 |
| **Anionic ampicillin** | 2 | -51.16 | -28.21 | 8.20 |

| rDock | Pose | Score | Inter | Intra | Van der Waals |
|---|---|---|---|---|---|
| **Zwitterionic ampicillin** | 3 | -24.474 | -14.473 | -2.350 | -16.323 |
| **Anionic ampicillin** | 4 | -21.235 | -13.457 | 0.870 | -16.301 |

| MM-PBSA | $\Delta G_{van\ der\ Waals}$ (kcal/mol) | $\Delta G_{van\ der\ Waals}$ (kcal/mol) | $\Delta G_{binding}$ (kcal/mol) | $\Delta G_{binding\ (\varepsilon=80)}$ (kcal/mol) |
|---|---|---|---|---|
| **Zwitterionic ampicillin** | -98.8199 | -21.2057 | -120.0256 | -9.5147 |
| **Anionic ampicillin** | -42.8651 | -22.8381 | -65.7032 | -5.6092 |

**FIGURE S7.** (Top) Docking poses of charged (yellow carbon atoms) and zwitterionic (orange carbon atoms) ampicillin to PorB. (Bottom) Tables showing docking scores of the binding poses for both GOLD and rDOCK docking softwares and MM-PBSA calculations performed on the selected docking poses to estimate the binding affinity.

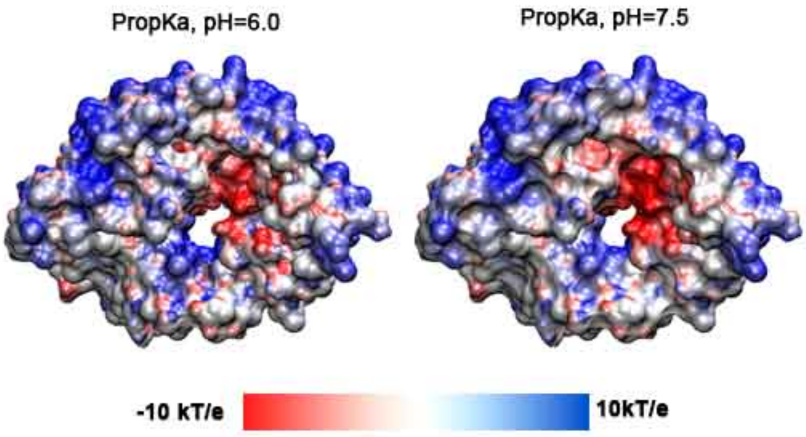


**FIGURE S8.** PropKa results on the PorB electrostatic surface potential. Top view of the PorB surface, coloured by its electrostatic potential at pH 6 and pH7.5. The colour bar corresponds to the electrostatic potential colour scheme (From red at -10kT/e to blue at 10kT/e).

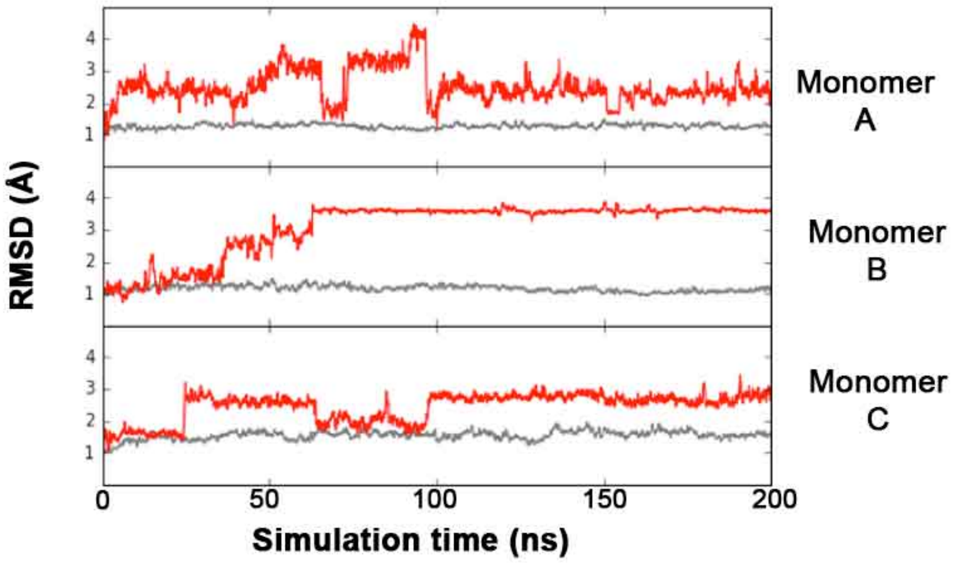


**FIGURE S9.** Root mean square deviation (RMSD) of unbiased molecular dynamics simulations of ampicillin-bound PorB systems. The RMSD is calculated for the Cα atoms (grey lines) of each PorB monomer and the heavy atoms of ampicillin (red lines) of the ampicillin-bound PorB simulations.

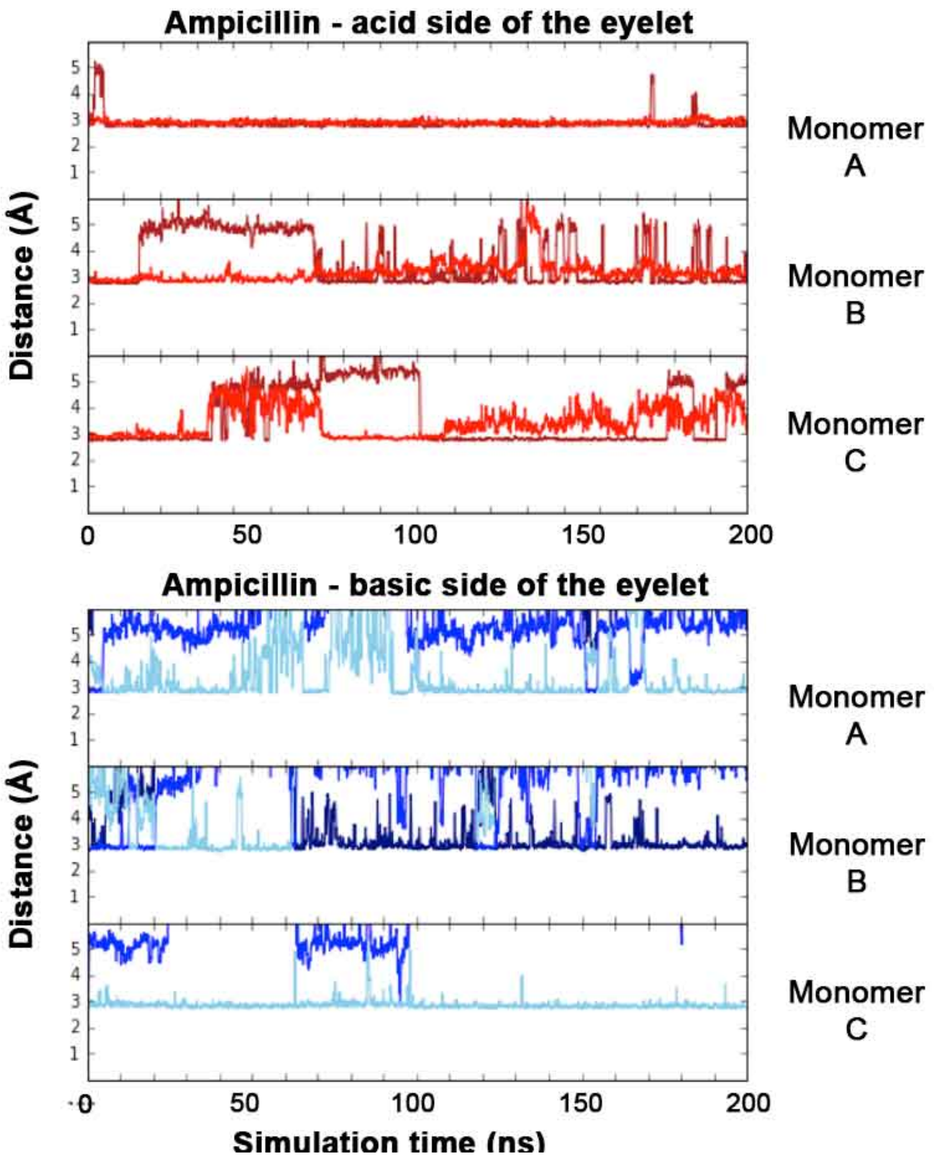


**FIGURE S10.** Interactions of the ampicillin molecule in the unbiased molecular dynamics simulations of ampicillin-bound PorB. (Top) Distances mapping the interactions of the ampicillin molecule with the acidic side (carbonyl atom of G112 in red lines and Glu116 in maroon lines) and, (bottom) the basic side (Lys64 in cyan lines, Lys100 in navy lines and Arg130 in blue lines) of the eyelet.

| | Apo PorB simulations (1M NaCl solution ). | | Apo PorB simulations (1M KCl solution ) | | Ampicillin bound PorB simulations (1M NaCl solution ) | |
|---|---|---|---|---|---|---|
| | **Positive voltages** | **Negative voltages** | **Positive voltages** | **Negative voltages** | **Positive voltages** | **Negative voltages** |
| **Final** | 1.80 ± 0.14 nS | 1.68 ± 0.17 nS | 1.75 ± 0.23 nS | 1.54 ±0.10 nS | 0.43 ± 0.12 nS | 0.15 ± 0.02 nS |
| **± 125 mV** | 1.58 ± 0.18 nS | 1.89 ± 0.21 nS | 1.62 ± 0.13 nS | 1.55 ± 0.07 nS | 0.34 ± 0.01 nS | 0.15 ± 0.03 nS |
| **± 350 mV** | 1.89 ± 0.05 nS | 1.59 ± 0.06 nS | 1.97± 0.06 nS | 1.53± 0.08 nS | 0.50 ± 0.06 nS | 0.14 ± 0.02 nS |
| **± 500 mV** | 1.94 ± 0.11 nS | 1.64 ± 0.11 nS | | | 0.54 ± 0.04 nS | 0.15 ± 0.01 nS |

**FIGURE S11.** Conductance values calculated from CompEl simulations of apo PorB systems (for both NaCl and KCl solutions) and ampicillin bound PorB systems at each voltage. Each data point corresponds to an overlapping 20ns-long window of the CompEl simulations.

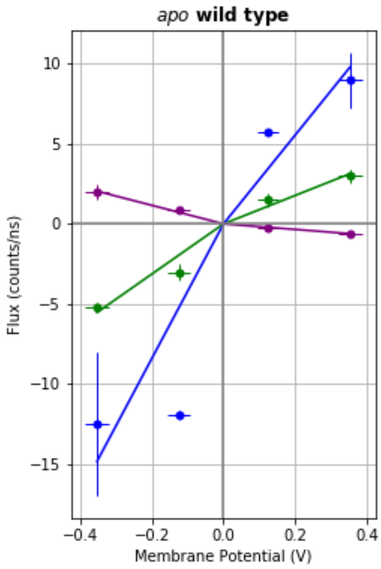


**FIGURE S12.** Flux of ions in the CompEl simulations using 1M KCl solution. The flux of $Cl^-$ (green), $Na^+$ ions (purple) and the flow of water molecules (blue) across the eyelet at negative and positive voltages is shown. Positive values of flux account for permeations from the extracellular compartment to the periplasmic compartment. Representative modes of ampicillin binding at positive (**B**) and negative voltages (**C**).

## References


(1) Angelova, M. I.; Dimitrov, D. S. Liposome Electro Formation. *Faraday Discuss. Chem. Soc* **1986**, *81*, 303–311.

(2) Pein, F.; Tecuapetla-Gómez, I.; Schütte, O. M.; Steinem, C.; Munk, A. Fully-Automatic Multiresolution Idealization for Filtered Ion Channel Recordings: Flickering Event Detection. *arXiv* **2017**, No. 1706.03671, Submitted.

(3) Hotz, T.; Schütte, O. M.; Sieling, H.; Polupanow, T.; Diederichsen, U.; Steinem, C.; Munk, A. Idealizing Ion Channel Recordings by a Jump Segmentation Multiresolution Filter. *IEEE Trans. Nanobioscience* **2013**, *12* (4), 376–386.

(4) Rabiner, L. R. A Tutorial on Hidden Markov Models and Selected Applications in Speech Recognition. *Proc. IEEE* **1989**, *77* (2), 257–286.

(5) Fletcher, R. *Practical Methods of Optimization*; (John Wiley and Sons, New York)., 1987.

(6) Jones, G.; Willett, P.; Glen, R. C.; Leach, A. R.; Taylor, R. Development and Validation of a Genetic Algorithm for Flexible Docking. *J. Mol. Biol.* **1997**, *267* (3), 727–748.

(7) Morley, S. D.; Afshar, M. Validation of an Empirical RNA-Ligand Scoring Function for Fast Flexible Docking Using RiboDock. *J. Comput. Aided. Mol. Des.* **2004**, *18* (3), 189–208.

(8) Barril, X.; Hubbard, R. E.; Morley, S. D. Virtual Screening in Structure-Based Drug Discovery. *Mini Rev. Med. Chem.* **2004**, *4* (7), 779–791.

(9) Kattner, C.; Zaucha, J.; Jaenecke, F.; Zachariae, U.; Tanabe, M. Identification of a Cation Transport Pathway in Neisseria Meningitidis PorB. *Proteins Struct. Funct. Bioinforma.* **2013**, *81* (5), 830–840.

(10) Homeyer, N.; Gohlke, H. Free Energy Calculations by the Molecular Mechanics Poisson-Boltzmann Surface Area Method. *Mol. Inform.* **2012**, *31* (2), 114–122.

(11) Homeyer, N.; Ioannidis, H.; Kolarov, F.; Gauglitz, G.; Zikos, C.; Kolocouris, A.; Gohlke, H. Interpreting Thermodynamic Profiles of Aminoadamantane Compounds Inhibiting the M2 Proton Channel of Influenza A by Free Energy Calculations. *J. Chem. Inf. Model.* **2016**, *56* (1), 110–126.

(12) Mulay, S. R.; Desai, J.; Kumar, S. V.; Eberhard, J. N.; Thomasova, D.; Romoli, S.; Grigorescu, M.; Kulkarni, O. P.; Popper, B.; Vielhauer, V.; et al. ProtSqueeze: Simple and Effective Automated Tool for Setting up Membrane Protein Simulations. *J. Chem. Inf. Model.* **2016**, *47* (5), 1986–1994.

(13) Wolf, M. G.; Hoefling, M.; Aponte-SantamaríA, C.; Grubmüller, H.; Groenhof, G. G-Membed: Efficient Insertion of a Membrane Protein into an Equilibrated Lipid Bilayer with Minimal Perturbation. *J. Comput. Chem.* **2010**, *31* (11), 2169–2174.

(14) Lindorff-Larsen, K.; Piana, S.; Palmo, K.; Maragakis, P.; Klepeis, J. L.; Dror, R. O.; Shaw, D. E. Improved Side-Chain Torsion Potentials for the Amber ff99SB Protein Force Field. *Proteins Struct. Funct. Bioinforma.* **2010**, *78* (8), 1950–1958.

(15) Cordomí, A.; Caltabiano, G.; Pardo, L. Membrane Protein Simulations Using AMBER Force Field and Berger Lipid Parameters. *J. Chem. Theory Comput.* **2012**, *8* (3), 948–958.

(16) Feenstra, K. A.; Hess, B.; Berendsen, H. J. C. Improving Efficiency of Large Time-Scale Molecular Dynamics Simulations of Hydrogen-Rich Systems. *J. Comput. Chem.* **1999**, *20* (8), 786—798.

(17) Lindahl, E.; Edholm, O. Mesoscopic Undulations and Thickness Fluctuations in Lipid Bilayers from Molecular Dynamics Simulations. *Biophys. J.* **2000**, *79* (1), 426–433.

(18) Mark, P.; Nilsson, L. Structure and Dynamics of the TIP3P, SPC, and SPC/E Water Models at 298 K. *J. Phys. Chem. A* **2001**, *105*, 9954–9960.

(19) Joung, I. S.; Cheatham, T. E. Determination of Alkali and Halide Monovalent Ion Parameters for Use in Explicitly Solvated Biomolecular Simulations. *J. Phys. Chem. B* **2008**, *112* (30), 9020–9041.

(20) Wang, J.; Wolf, R. M.; Caldwell, J. W.; Kollman, P. A.; Case, D. A. Development and Testing of a General Amber Force Field. *J. Comput. Chem.* **2004**, *25*, 1157–1174.

(21) Wang, J.; Cieplak, P.; Kollman, P. A. How Well Does a Restrained Electrostatic Potential (RESP) Model Perform in Calculating Conformational Energies of Organic and Biological Molecules? *J. Comput. Chem.* **2000**, *21* (12), 1049–1074.

(22) Case; Case, D. A.; Darden, T. A.; Cheatham, T. E.; Simmerling, C. L.; Wang, J.; Duke, R. E.; Luo, R.; Walker, R. C.; Zhang, W.; et al. *AMBER 12*; University of California, San Francisco: San Francisco, 2012.

(23) Abraham, M. J.; Murtola, T.; Schulz, R.; Páall, S.; Smith, J. C.; Hess, B.; Lindah, E. Gromacs: High Performance Molecular Simulations through Multi-Level Parallelism from Laptops to Supercomputers. *SoftwareX* **2015**, *1–2*, 19–25.

(24) Bussi, G.; Donadio, D.; Parrinello, M. Canonical Sampling through Velocity Rescaling. *J. Chem. Phys.* **2007**, *126* (1).

(25) Berendsen, H. J. C.; Postma, J. P. M.; van Gunsteren, W. F.; DiNola, a; Haak, J. R. Molecular Dynamics with Coupling to an External Bath. *J. Chem. Phys.* **1984**, *81* (8), 3684–3690.

(26) Darden, T.; York, D.; Pedersen, L. Particle Mesh Ewald: An N log(N) Method for Ewald Sums in Large Systems. *J. Chem. Phys.* **1993**, *98* (12), 10089.

(27) Hess, B.; Bekker, H.; Berendsen, H. J. C.; Fraaije, J. G. E. M. LINCS: A Linear Constraint Solver for Molecular Simulations. *J. Comput. Chem.* **1997**, *18* (12), 1463–1472.

(28) Miyamoto, S.; Kollman, P. A. SETTLE: An Analytical Version of the SHAKE and RATTLE Algorithm for Rigid Water Models. *J. Comput. Chem.* **1992**, *13* (8), 952–962.

(29) Kutzner, C.; Grubmüller, H.; De Groot, B. L.; Zachariae, U. Computational Electrophysiology: The Molecular Dynamics of Ion Channel Permeation and Selectivity in Atomistic Detail. *Biophys. J.* **2011**, *101* (4), 809–817.

(30) Michaud-Agrawal, N.; Denning, E. J.; Woolf, T. B.; Beckstein, O. MDAnalysis: A Toolkit for the Analysis of Molecular Dynamics Simulations. *J. Comput. Chem.* **2011**, *32* (10), 2319–2327.

(31) McGibbon, R. T.; Beauchamp, K. A.; Harrigan, M. P.; Klein, C.; Swails, J. M.; Hernández, C. X.; Schwantes, C. R.; Wang, L. P.; Lane, T. J.; Pande, V. S. MDTraj: A Modern Open Library for the Analysis of Molecular Dynamics Trajectories. *Biophys. J.* **2015**, *109* (8), 1528–1532.
